# Functional Characteristics and Mechanical Performance of PCU Composites for Knee Meniscus Replacement

**DOI:** 10.3390/ma13081886

**Published:** 2020-04-17

**Authors:** Adijat Omowumi Inyang, Christopher Leonard Vaughan

**Affiliations:** Division of Biomedical Engineering, Human Biology Department, Faculty of Health Sciences, University of Cape Town, Anzio Road, Observatory, Cape Town 7925, South Africa; kit.vaughan@uct.ac.za

**Keywords:** composite, fibers, polycarbonate-urethane, meniscal replacement, mechanical properties, meniscus

## Abstract

The potential use of fiber-reinforced based polycarbonate-urethanes (PCUs) as candidate meniscal substitutes was investigated in this study. Mechanical test pieces were designed and fabricated using a compression molding technique. Ultra-High Molecular Weight Polyethylene (UHMWPE) fibers were impregnated into PCU matrices, and their mechanical and microstructural properties evaluated. In particular, the tensile moduli of the PCUs were found unsuitable, since they were comparatively lower than that of the meniscus, and may not be able to replicate the inherent role of the meniscus effectively. However, the inclusion of fibers produced a substantial increment in the tensile modulus, to a value within a close range measured for meniscus tissues. Increments of up to 227% were calculated with a PCU fiber reinforcement composite. The embedded fibers in the PCU composites enhanced the fracture mechanisms by preventing the brittle failure and plastic deformation exhibited in fractured PCUs. The behavior of the composites in compression varied with respect to the PCU matrix materials. The mechanical characteristics demonstrated by the developed PCU composites suggest that fiber reinforcements have a considerable potential to duplicate the distinct and multifaceted biomechanical roles of the meniscus.

## 1. Introduction

The meniscus is a complex and vital biomechanical fibrocartilaginous tissue in the knee joint. The menisci are important structures, as they have a participatory role in shock absorption, joint lubrication and joint congruency [1,2,3,4]. As a result of a large amount of force borne by the meniscus, it is frequently subjected to tear, and it wears out with time. Meniscal tears have been extensively reported as one of the most recurrent knee injuries [5,6,7,8,9]. Damage to the meniscus affects its load sharing and distribution roles performed in the knee, which has been linked to the degeneration of the articular cartilage and a high risk of the devastating disease, osteoarthritis [9,10,11]. 

Meniscus removal has considerable repercussions for the joint, as it causes abnormal contact pressures, resulting in joint degeneration [12]. As a result of the consequences of meniscectomy, alternative measures have involved the repair or replacement of the meniscus. However, limited success has been achieved with available options owing to various limitations, such as repair only being possible when the tear occurs in the vascularized area, which does not heal well due to shortage of blood supply [13]. Allografts are alternatives for replacing the meniscus of younger patients with a meniscectomized knee joint [14]. Although allografts have acceptable clinical outcomes, the long-term examinations revealed debatable protective effects to the cartilage [15]. Besides, meniscal allografts have difficulty with remodeling and lack adequate strength [12]. Consequently, allografts cannot be an absolute cure for post-meniscectomy pain [14]. On the other hand, meniscal replacements are biomechanically suitable, and have a distinct advantage of acting as substitutes in the cases of multifaceted tears, as well as alleviating the excruciating pain related to meniscus damage [16].

Different replacements have been sought for the meniscus using either synthetic scaffolds [17,18], natural meniscal tissues or composite materials [19,20,21,22,23,24]. Amongst the first meniscus replacements developed were permanent meniscal substitutes made from teflon and dacron [11,25,26]. These alternatives were found incompatible after in-vivo testing in rabbits as particles resulting from wear were deposited on the implant. Besides, mechanical integrity was compromised. Similarly, researchers have worked on scaffolds of poly(lactic-co-glycolic acid) embedded with polyglycolic acid fiber [27]. Although the in-vivo studies showed that the scaffolds produced meniscus-resembling tissue after ten weeks’ implantation, mechanically the modulus of the tissue was inadequate, as it was not comparable with that of the native meniscus. Another development was a composite of hyaluronic acid and the polycaprolactone matrix with poly-lactic acid reinforcement fibers. The in-vivo studies established that the composite materials support meniscal tissue growth devoid of any adverse effect on the cartilage after implantation, but there was failure resulting from implant extrusion [22,28].

Balint et al. [29] used a different approach in developing a total meniscal substitute where a porous scaffold of collagen-hyaluronan matrix with degradable poly (desaminotyrosyl-tyrosine dodecyl ester dodecanoate) reinforcement fibers was studied. These scaffolds proved to be successful, with considerable mechanical properties suitable as meniscal substitutes; however, implant extrusion remains a challenge [30,31]. The use of polyvinyl alcohol (PVA) hydrogel as a choice material for permanent meniscus replacement has been investigated [32,33]. The study showed the inability of the implant to protect the articular cartilage, and the authors concluded that the failure could be due to size incongruity and inefficient fixation [34].

Studies have explored the possibility of incorporating Ultra High Molecular Weight Polyethylene (UHMWPE) fibers into different matrices, such as poly (vinyl alcohol) hydrogels and polycarbonate-urethane [35,36]. The former group fabricated meniscal substitutes that showed promising mechanical properties and manufacturability. They reported limitations, including delamination and implant extrusion [37,38]. In the latter study, the mechanical behavior of the meniscal prosthesis was determined mathematically and experimentally using a knee model subjected to compressive loading. However, it is indispensable to fully characterize the mechanical properties of the developed composite material in-vitro. Moreover, the finite element model is an approximation of the numerical model [39].

Although several researchers have worked on the development of meniscal substitutes, most of their attempts have been focused on investigating the biological characteristics of the implants from a tissue engineering perspective. While these factors are critical for body replacement parts, the mechanical properties of the developed implants are not given adequate consideration. This gap is evident from the failure reported in literature [40,41,42]. As a result, there is a dearth of information describing the mechanical requirements and functional performance of replacement for the meniscal tissue. Due to the load-bearing capabilities of the meniscus and its exposure to millions of cycles on an annual basis [43,44], the importance of evaluating the mechanical characteristics of the implant material intended to replace the meniscus cannot be overemphasized, since this will provide critical information, such as safety before implantation. Furthermore, current failures associated with existing permanent synthetic meniscal implants, such as insufficient strength, durability, dislocation, wear and fracture [40] further buttressed the need for assessing the mechanical behavior of a material proposed to replace the meniscus. Thus, the choice of appropriate materials for design, the geometry and the mechanical attributes, could produce a suitable candidate for replacing the meniscus.

The meniscus, being a bundle of cartilaginous tissue, has a complex make-up of material properties which vary with location as well as direction [45]. Its peculiar and particular roles emanate from its unique chemical, physical and biomechanical composition, as well as its distinctive structural architecture [46]. 

Therefore, in order to restore the biomechanical tasks of a worn-out meniscus, it is important to circumspectly select a replacement material having biomechanical properties as close as possible to those of a native meniscus. Though the meniscus demonstrates site-dependent properties, both in tension and compression, it is anticipated that a composite material can be precisely fitted to duplicate these properties, and hence replace the meniscus. Thus, an isotropic matrix material may not be able to produce the inhomogeneous and anisotropic characteristics exhibited by the natural meniscus [45], but fiber-reinforced polycarbonate-urethanes (PCU) could be customized and adapted based on a suitable choice of reinforcement fiber and its orientation within the PCU matrix. Additionally, the use of fiber-reinforced composite will enable the reinforcing fibers to act as a channel for attaching the implant to the joint capsule to cater for dislocation during motion. Incorporating fibers with high strength into soft polymeric matrices to engineer synthetic meniscal replacement has not been extensively explored. Consequently, this work aimed at developing a structured, tailor-made, PCU-reinforced UHMWPE (referred to as PE) composite as a meniscal substitute. Several surface engineering approaches have been introduced in order to modify the surface structure of UHMWPE for improved biomedical applications. These methods, which include plasma techniques [47], laser surface modification [48] and incorporating particulate or fibrous reinforcements [49,50], have been used to enhance the mechanical, tribological and biological properties of UHMWPE substrates.

Medical grade polyurethanes have been widely promoted for biomedical applications [51]. In particular, the use of PCU has drawn considerable attention in the orthopedic device industry as a result of their excellent mechanical properties, biostability and biocompatibility [52]. PCUs have been extensively utilized in vascular grafts, stents, catheters, pacemaker leads and artificial heart valves [53]. Specifically, bionate thermoplastic PCU, commercially produced by DSM PTG (Berkeley, CA, USA), has been of great interest in the field of orthopedics, because of its outstanding load-bearing properties and excellent wear resistance, which enables it to overcome the setback of osteolysis. Its superior characteristics have made it an excellent material selected for hip and knee joint prostheses, prosthetic spinal discs and the shoulder joint system [54]. Besides, they offer long term durability and resistance to hydrolytic degradation, making them outstanding for in-vivo orthopedic applications [55]. The use of PCU for meniscus replacement stemmed from its unique weight-bearing capabilities, the ability to withstand intense forces within the knee joint [56], and an ease of lubrication due to its hydrophilic nature. Moreover, it proffers low friction properties [57,58,59] to promote movement within the meniscal compartment, while withstanding repeated stresses from the femoral condyle during flexion and extension motions.

Therefore, the overall goal of this study was to develop a meniscal substitute with mechanical properties closely matching those of the native meniscus. For that reason, test pieces of PCU-PE composites were designed, fabricated and evaluated to determine their suitability as a replacement for the meniscus capable of replicating the closest possible mechanical behavior of the native meniscal tissue. The mechanical test pieces were made up of longitudinally-arranged fibers, such as to duplicate the orientation of the circumferential collagen fiber existing in the human meniscus. The effects of the reinforcing fibers on the mechanical properties of the PCU were investigated, and the composites examined and appraised as meniscal implants. The fibers were able to provide a substantial increase in the stiffness of the PCU matrix and enhance the fatigue and abrasion resistance for long term implantation in the knee joint capsule.

## 2. Materials and Methods

### 2.1. Materials and Processing

Biomedical Bionate 80A and 90A polycarbonate urethane (PCU) from DSM (PTG, Berkeley, CA, USA) were used as the matrices, and the reinforcing fibers were Ultra High Molecular Weight Polyethylene (UHMWPE) continuous strand fibers, Dyneema Purity^®^ UG from DSM (designated as PE). Initial drying of the PCU pellets was done at 100 °C for 14 h in a vacuum oven, as stipulated by the supplier and established by Geary et al. [55] as the optimal drying conditions for PCUs to reduce the moisture content to about 0.01%. The intrinsic properties of the PCUs and the reinforcing fibers are detailed in Table 1, as stated in the supplier’s data sheets.

### 2.2. Composite Preparation

**Figure 1:**
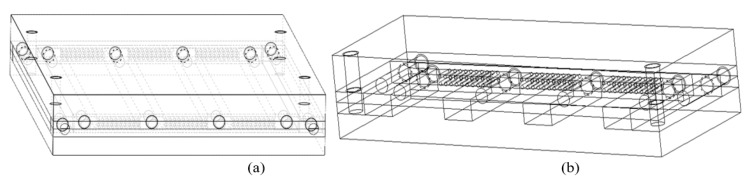
The fabricated mold for the test pieces showing (**a**) The entire mold assemblage (**b**) The fiber holes within the mold.

A stainless-steel, tailor-made mold was fabricated to produce the composite mechanical test samples (Figure 1). The mold, which encloses the fiber, consists of different parts that are assembled to provide the facile removal of cured samples. The composite samples were formulated using a 5% fiber volume fraction. The percentage of the various constituents was based on a computation of fiber diameter, fiber length and the number of fibers, as discussed in our previous work [60]. The composite material was prepared using the combinations of the different PCUs and the reinforcement fibers. The specimen types and their constituents are described in Table 2.

The reinforcing fibers were arranged prior to the composite preparation and inflexibly gripped within the mold, such that they were embedded equidistantly within the PCU matrix. After that, the PCU pellets were dispersed to fill the mold. Subsequently, the test pieces were compacted in a pre-heated, custom-built hot press at a pressure of 15 MPa. The PCU and their reinforced samples were cured at 190 °C for MX1 and MP1 samples, and 200 °C for MX2 and MP2 samples for about 10 min. Preliminary experiments were performed to ascertain these molding temperatures, and the amount of pellets required for optimal sample production, since the polymeric matrices and the fibers have varying melting temperatures. The mold was then cooled to room temperature before taking out the samples.

### 2.3. Mechanical Evaluation

**Figure 2:**
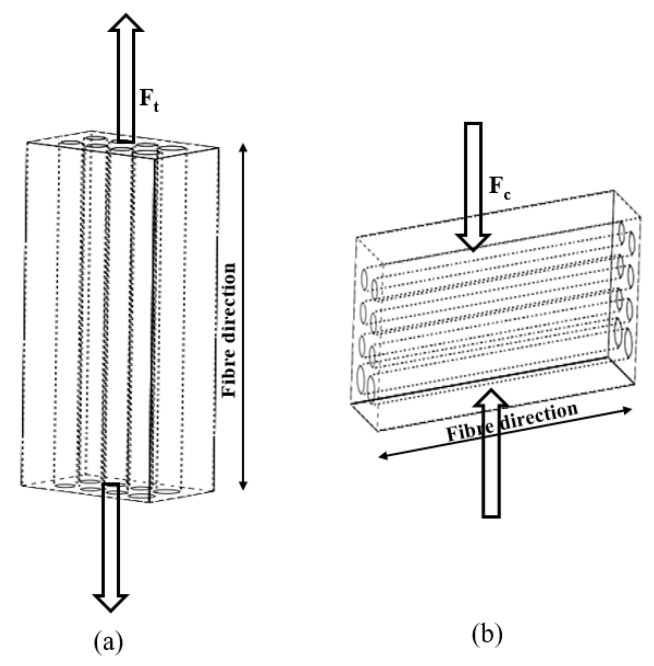
Graphical representation of the mechanical test pieces describing the fiber arrangement and direction for (**a**) tensile testing, F_t_ and (**b**) compression testing, F_c_. The diagrams are not drawn to scale.

The mechanical test samples were tested for tensile, F_t_, and compressive, F_c_, properties (Figure 2) using a Zwick/Roell 1484 Material Testing Machine (Zwick GmbH & Co. KG, Ulm, Germany). Each rectangular cuboid tensile specimen of 70 × 19 × 6 mm, was tested at a crosshead speed of 12 mm/min (Figure 2a). Cubic specimens of 6 × 6 × 6 mm were tested for compression, F_c_, at a crosshead speed of 5 mm/min (Figure 2b). The moduli for the tensile and compressive tests were calculated from the slope of the linear region of the stress–strain plots. The tensile modulus was taken as the slope of a linear curve fit between 0% to 5% strain, while the compressive modulus was between 2% and 8% strain. These tests were also performed on 100% virgin PCU samples. Three specimens were analyzed in each of the tests. All the results were computed as mean ± standard deviation, and with Excel software (Microsoft, Washington, USA), the unpaired Student’s t-test was used to assess the data statistically for significant difference at *p* < 0.05.

### 2.4. Microstructural Analysis

The arrangements and alignments of the fibers in the matrices were studied using a Wild M400 photomacroscope (Wild Heerbrugg, Gais, Switzerland). In addition, the Nova NanoSem 230 scanning electron microscope (SEM) (FEI, Holland, Netherlands) was used to examine the morphology of the PCUs and their composites, and to investigate the fractured surfaces of the tested samples after failure during the mechanical testing.

## 3. Results and Discussion

### 3.1. Microstructural Characterization

In order to study the distribution of fibers in polymeric matrices, a useful approach is to examine their fractured surfaces after mechanical testing through scanning electron microscopy. An accurate and detailed investigation of the fractured surfaces provides information on the nature of the interfacial bonding existing within the fiber–matrix interface. It explains the phenomenon taking place during the deformation process. Also, the knowledge of the failure mechanisms of the PCU and their composites is crucial in evaluating their long-term mechanical reliability for the proposed applications. 

**Figure 3:**
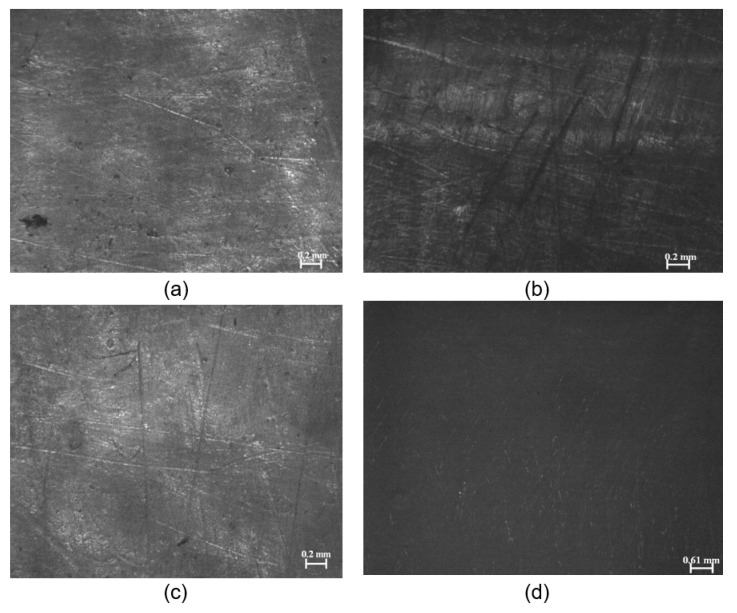
Photomicrographs showing the molded samples of (**a**) MX1 (**b**) MX2 (**c**) MP1 (**d**) MP2.

Consequently, the surface morphology of the fabricated samples was microscopically viewed to investigate the effects of the reinforcement fibers. The observed micrographs of the PCU matrices and their reinforced composites from the light microscope are presented in Figure 3a–d. The photomicrographs showed that the surface of the reinforced matrices appeared relatively smoother and cleaner with minimal surface imperfections, compared to the as-molded polymeric matrices. 

**Figure 4:**
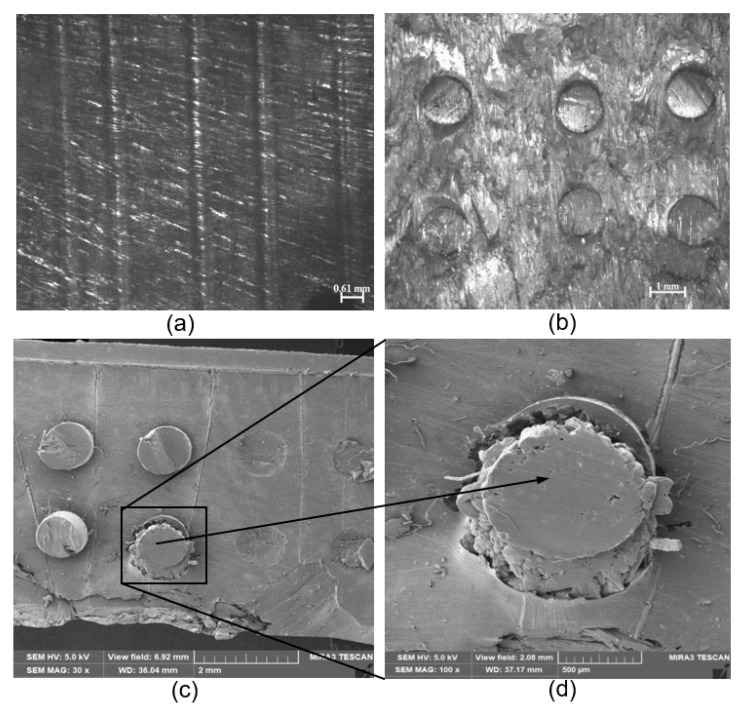
Representative photomicrographs showing (**a**) a transverse sectional view of the arrangement and alignment of the fibers within the composite (**b**) a cross-sectional view of the arrangement and alignment of the fibers within the composite. Scanning electron microscopy showing a cross-section of (**c**) the distribution of the PE fibers within the PCU (**d**) a magnified view of the PE fiber embedded in the PCU matrix.

The fibers were almost evenly distributed within the PCU matrix and at equal distances to one another (Figure 4a). A micrograph of the cross-sectional view of the sample observed from the same microscope corroborated the previous observation (Figure 4b).

Furthermore, the SEM examination, as indicated in Figure 4c, ascertained the uniform distribution of the fiber orientation. The fiber holes, as seen in Figure 4c, were respectively arranged parallel to one another. At higher magnification, the PE fiber was observed to be thoroughly embedded in the polymeric PCU matrix with the surrounding formed ring of PCU-PE fiber interface (Figure 4d).

**Figure 5:**
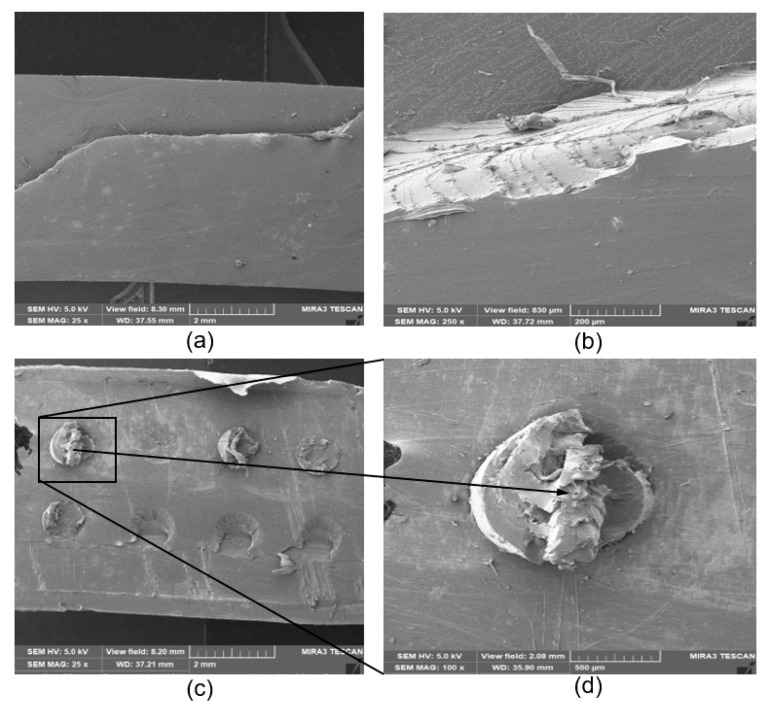
Scanning electron microscopy (SEM) showing fractured surfaces of the samples under tensile loading for (**a**) B8 matrix (**b**) cross-sectional view of the fractured surface of B8 matrix. (**c**) PE-PCU composite along the fiber direction B8 composite (**d**) a closer view of the fractured PE fiber.

The fracture behavior of the as-molded PCU samples is shown in Figure 5a,b. The illustrative SEM images showed a sharp, rough and angular fractured surface of the PCU polymer. This surface is characterized by striations suggesting the direction of the crack propagation. The large surface area characterized by smooth regions reveals fast brittle fracture, which is typical of elastomeric PCUs [61,62]. The minimal protrusions disintegrating at the fractured side on the relatively smooth surface (Figure 5b), suggest somewhat the plastic deformation of the PCU polymeric matrix.

On the other hand, PCU-PE composites showed a smooth surface with the fibers firmly held intact in the matrix (Figure 5c,d). A magnified view of the fiber showed an irregular, well-bonded PCU-PE surface. A comparison of the fiber arrangement in the PCU matrix before and after tensile loading gives the information on what ensued when the tensile load was applied (Figure 4b and Figure 5c). SEM images showed the fractured surfaces of the composite material under tensile loading along the fiber direction, with none of the fibers displaced from their average initial positions (Figure 5c). 

The SEM images of the PCU-PE composites after the tensile loading showed no evidence of PE fiber pull-out from the PCU matrix, as no void was observed on the microscopically examined surfaces (Figure 5c,d). This observation suggests that there is a strong interfacial bonding that exists between the PCU and the PE fibers. The strong adhesion transmits the applied load from the PCU matrix to the PE fibers, producing a significant rise in the overall mechanical characteristics of the composite material. The existence of the PCU matrix on the surface of the fiber shows there is a strong interaction between the fibers and the PCU, as seen in Figure 5d. The interfacial bonding strength has a corresponding positive effect on the mechanical properties of composite materials [63], hence the increased stiffness observed in the reinforced PCU compared to the virgin PCUs.

### 3.2. Mechanical Properties

**Figure 6:**
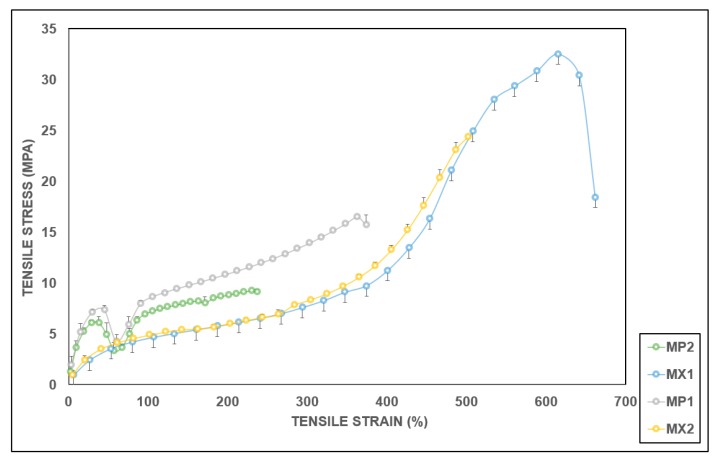
Average tensile stress–strain plots for the PCUs and the PCU-PE composites.

**Figure 7:**
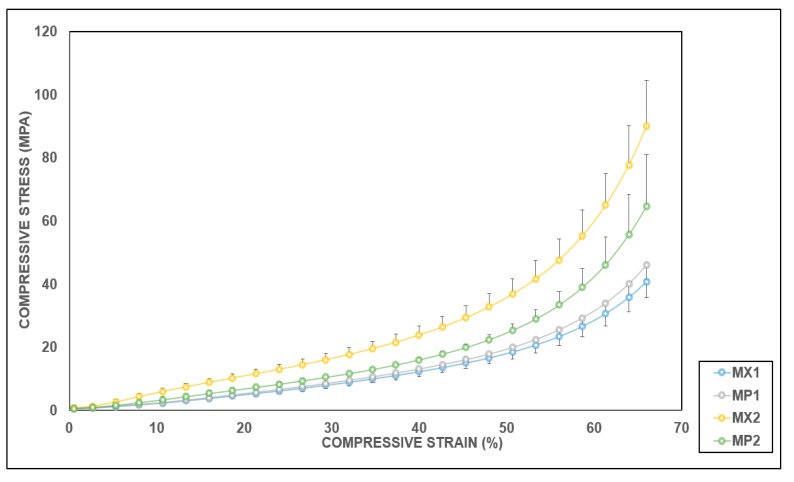
Average compressive stress–strain plots for the PCUs and the PCU-PE composites.

The stress–strain graphs for the average values of the tested samples for both tension and compression tests were plotted in Figure 6 and Figure 7, respectively. These curves exhibited a linear pattern at low strains. Subsequently, a considerable change followed in the slope presenting a nonlinear behavior that continued until the tested samples began to fail. Both MX1 and MX2 demonstrated typical elastomeric stress–strain behavior [55,64]. The PCUs were not as stiff as their fiber-reinforced composites, which indicates that the increase is a function of the stiffness of both the matrix and the interspersed fibers. 

**Figure 8:**
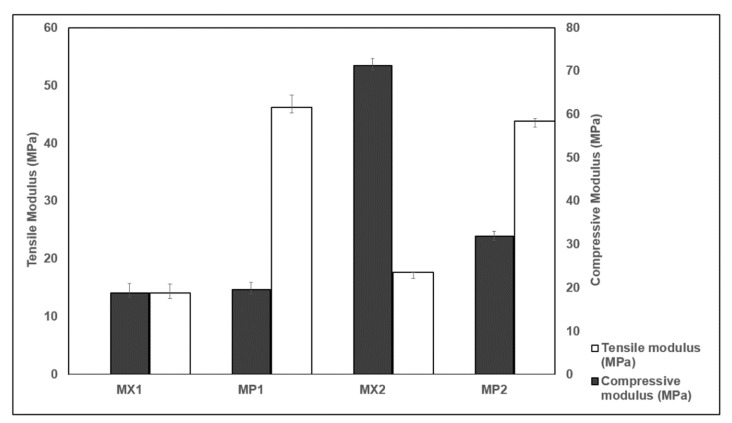
Tensile and compressive properties of the PCU-PE composites compared with their unreinforced matrices.

A meniscal substitute must be able to perform similar functions as the natural weight-bearing meniscal structure. To this end, the tensile and compressive properties of the PCUs and their fiber-reinforced composites have been evaluated relative to the meniscus tissue to optimize them as potential meniscal replacements. The empirically calculated tensile moduli for the PCU matrices were rather disparate to those of the supplier (Table 1). These discrepancies could be as a result of the manufacturer’s test conducted at 37 °C conditioned in water, since PCU properties are temperature-dependent (DSM-PTG). Besides, their samples were annealed for 24 h at 70 °C before testing. Generally, the moduli of all reinforced specimens were higher than their unreinforced counterparts. The tensile modulus of the meniscus is site-dependent, and hence varies relative to the area and direction. The circumferential tensile modulus of the human meniscus varies between approximately 58 MPa and 295 MPa, while the radial tensile modulus varies between approximately 3 MPa and 60 MPa [65,66,67,68]. Therefore, it is expedient that a meniscal device should possess a circumferential tensile modulus of at least 58 MPa in order to prevent deformation, as well as implant extrusion maximally. Both of the PCUs studied offer much lower stiffness (Figure 8). Thus, they will not appropriately perform the rigorous tasks that the meniscal tissue is subjected to on a routine basis. Reinforcing the soft polymeric matrices with durable, high-performance fibers such as PE could therefore possibly construct a composite material which is biomechanically acceptable to replace the worn-out meniscus.

The tensile moduli of composites of MX1 and MX2 increased appreciably with the incorporation of the PE fibers. MP1 and MP2 exhibited higher stiffness than the PCU matrices, as the fibers significantly (*p* < 0.05) enhanced the tensile properties of the PCUs. Percentage increases of 227% and 148% were obtained for MX1 and MX2, respectively. Interestingly, a similar trend was observed in the tensile characteristics of the curves of the following pairs: MX1 and MX2, and also MP1 and MP2, which revealed similar patterns (Figure 6 and Figure 7). This suggests that the behavior of the fiber is similar, irrespective of the matrix material, further establishing the role of the fibers in influencing the overall performance of a composite material. The function of the fibers could be further understood by the details seen in the micrographs (Figure 5c,d), where the fibers were oriented, and remained in their original positions as they “fought” to withstand the applied tensile load.

The compression behavior of the implant device is pivotal to its overall performance, since the meniscus provides support for knee joint stability [69]. In addition, the compressive modulus is of great significance, as it resists the high stresses and transmits the compression loads exerted by the femur on the tibia.

Unlike the tensile moduli, there was a variation in the changes observed in the compressive moduli with the inclusion of fibers in the PCUs compared to their unreinforced counterparts. All the specimen types behave similarly in compression irrespective of their constituent’s composition. The menisci are reported to transmit approximately 50%, and about 85%, of the compressive forces exerted in the knee in extension and 90° in flexion, respectively [70,71]. This role is made possible by the distinctive arrangement of the collagen fibers, which withstand the high stresses produced during the load-bearing. Consequently, a meniscal replacement must be able to reproduce the aforementioned characteristics and peculiarities associated with the native meniscus. An extensive range of compressive moduli values has been published, in which the variation was controlled by strain and loading rates, as well as the type of test conducted. The aggregate compressive modulus varying between 0.10 and 1.13 MPa has been reported for the native meniscus [65,72,73,74]. In this study, the minimum compressive modulus was recorded for MP1 composite with a 4% rise with the addition of fibers, while MP2 produced a considerable 55% reduction with the inclusion of fibers. The addition of the PE fibers to MX2 were found to be statistically significant in compression. The difference observed in the compressive moduli of MX1 and MX2, and their composites, showed the influence of fiber reinforcements in the compressive properties of the PCU matrices. Although the values obtained are not comparable to those reported for the human meniscus, a higher compressive modulus will be tolerable and acceptable for a meniscal substitute, since some polymers and metals whose compressive moduli are much higher have been utilized as spacers in knee replacement devices [75]. The mechanical characteristics of composites are ultimately determined by the interfacial bond strength between the fiber and the matrix, which is dependent upon the type, shape, orientation and texture of the fiber surface. Consequently, the PCU-reinforced composites can be customized to mimic the desired properties of the native meniscus.

While the PCU composites exhibited excellent mechanical properties in tension and compression, MP1 produced a relatively high tensile modulus close to the natural meniscal range of values and a lower desirable compressive modulus. This comparatively low stiffness could be attributed to a drawback of the fibers encountered during the processing of the composite samples. The PE molecules tend to exhibit some form of relaxation and reorientation, even below the melting point. Besides, under rigorous loading conditions, the fiber molecules can slide, forming new arrangements, which phenomenon in the long run elongates the fiber, thus causing a reduction in tension leading to failure. These molecular changes may trigger a loss in the tensile properties depending on factors like temperature, time and loading conditions (Dyneema Purity^®^ UG, DSM). Therefore, it is anticipated that PE fibers with a higher melting point than the MX1 will produce exceptional results, both in tension and compression. During repetitive tensile stress, failure of the PCU composite can result from fiber fracture or fiber-matrix interfacial debonding. In such a case, the fibers can be detached from the matrix. When the applied tensile load extends the matrix beyond the fibers, the PCU composite will withstand shear at the fiber–matrix interface, which could cause it to fracture [76].

When the meniscus is subjected to an axially compressive force, the load is distributed over its surface area. Due to the meniscal structure, the transmitted force tends to cause the tissue to extrude radially. This structural malalignment is opposed by the hoop stresses generated in the circumferential collagen fibers [2,3]. These tensile stresses, developed within the meniscus during loading, control their function, and are responsible for failure [67]. The ultimate tensile stress of the native meniscus varies with respect to region. The average maximum stress has been reported to be 18.8 MPa, 17.6 MPa and approximately 4 MPa for lateral, medial and radial meniscal tissue, respectively [66]. Consequently, an average ultimate tensile stress of at least 18.8 MPa would be ideal for a meniscal substitute material. Of the composites, the MP1 performs extremely well within this limit.

**Figure 9:**
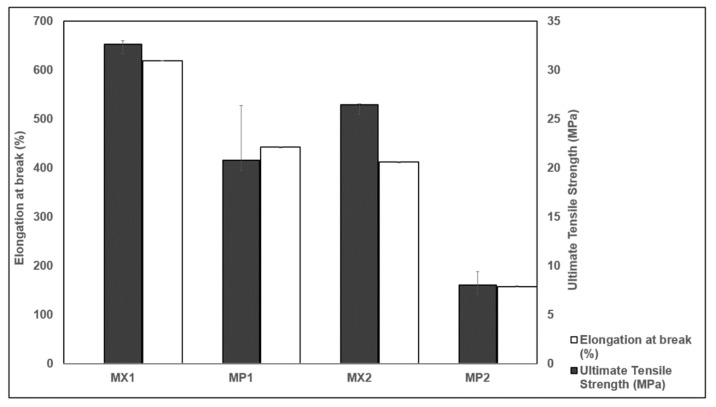
Elongation at break and ultimate tensile strength of the PCU-PE composites compared with their unreinforced matrices.

The ultimate tensile strengths of the PCUs were lower than those from the manufacturer’s data sheets (Table 1 and Figure 9). This could be as a result of the disparities in the shape and size of the tested samples, and also the testing technique employed. The average ultimate tensile strength of both composites decreased with fiber reinforcement, and reductions of 36% and 70% were calculated for both MP1 and MP2, respectively. These decreases could be because of the integral sample property, following that tensile strength is a characteristic of both the component materials and the composite samples under examination. Moreover, the tensile properties of polymeric matrix composites are considerably dependent on several factors like the matrix–fiber interface, geometry, distribution and micromechanical deformation [77].

The elongation at break decreased when the PE fibers were incorporated into the PCU matrices (Figure 9). Elongation at break is a measure of the ductility of a polymeric material, and indicates its ability to resist changes in shape without failing. With PE fibers-incorporated PCU composites, the elongation at break for MX1 and MX2 were reduced by about 28.5% and 61.8%, respectively. Fiber reinforcement of polymeric matrices increases the stiffness and toughness of composites. As the stiffness increases (Figure 6), there is a decrease in the ductility of the composite material, and hence the calculated elongation at break. Previous work has reported similar occurrences [78,79,80]. The value reported in the manufacturer’s datasheet for the PCU suggests that it can attain percentage elongation as high as 531%. At the same time, results from this study showed a higher maximum strain to failure of 619%. The changes in the percentage fracture strains could be due to factors like better surface finish and the firmness of the tensile grips. The wide-ranging tensile and compressive characteristics of the native meniscus complicates the development of a comparable meniscal implant. 

Since the functioning of the meniscus is critically reliant on its multiplex shape and structure, it is anticipated that a “true” reflection of the mechanical properties of the fabricated composites could be appropriately exhibited when the prosthesis is designed and manufactured to reproduce the structural configuration of the normal meniscus.

## 4. Conclusions

The mechanical performance of the PCU matrices determined from this study for the PCU matrices showed they are inadequate, and cannot replace nor sufficiently perform the load-bearing functions of the meniscus. In general, the effect of the fiber reinforcement was favorable, as the tensile modulus was significantly raised to a value within an acceptable tensile modulus of the human meniscus. The results from this study demonstrate that the PCUs can be customized to fit that of the meniscal tissue, by methodically implanting circumferential fibers into the PCU matrix to obtain a meniscal device with desirable mechanical properties. These results visibly revealed the positive effects of the reinforcing fibers.

Furthermore, the microstructural analysis revealed the failure mechanisms during mechanical testing. The embedded fibers in the PCU-PE composites prevented the brittle failure and plastic deformation exhibited in the fractured PCUs. The excellent interfacial bonding strength within the PCU-PE composites produced a corresponding positive effect on the mechanical properties of composite materials. Hence the increased stiffness observed in the reinforced PCUs compared to the virgin PCUs.

This work provides an insight into the mechanical and microstructural performance exhibited by the PCUs and their composites, hence their suitability for artificial bearing surfaces. Further characterization of the composite materials is required to determine their tribological behavior as a meniscal replacement.

## Figures and Tables

**Table 1:** Characteristic properties of the polycarbonate-urethanes (PCUs) and PCU-reinforced Ultra-High Molecular Weight Polyethylene (PE) fiber.

Material Property	80A	90A	PE Fiber
Tear Strength (kN/m)	64.90	96.40	—
Ultimate Tensile Strength (MPa)	46.64	55.11	—
Density (kg/m^3^)	1190	1200	960
Elastic Modulus (MPa)	12	29	126,000
Melting temperature (°C)	—	—	220
Diameter (mm)	—	—	0.31
Elongation at break (%)	531	406	—

**Table 2:** Material constituents for the specimen types.

Matrix	Fiber	Specimen Type
Bionate 80A	—	MX1
	PE	MP1
Bionate 90A	—	MX2
	PE	MP2
